# Predictive value of the cardiogenic shock working group-modified SCAI criteria in early-stage heart failure-related cardiogenic shock

**DOI:** 10.1016/j.ijcha.2025.101776

**Published:** 2025-08-26

**Authors:** Tijmen H. Ris, Morsal Atazadah, Roel Hoek, Jeroen Hoogland, Tim Balthazar, Federico Pappalardo, Paul Knaapen, Mariëlle C. van de Veerdonk, Alexander Nap

**Affiliations:** a Department of Cardiology, Amsterdam University Medical Center, Vrije Universiteit Amsterdam, Amsterdam Cardiovascular Sciences, Amsterdam, the Netherlands; b Department of Epidemiology & Data Science, Amsterdam University Medical Center, Vrije Universiteit Amsterdam, Amsterdam Cardiovascular Sciences, Amsterdam, the Netherlands; c Department of Cardiovascular Diseases, University Hospital Brussels, Jette, Belgium; d Department of Intensive Care, University Hospital Brussels, Jette, Belgium; e Kore University, Enna and Policlinico Centro Cuore GB Morgagni, Catania, Italy

**Keywords:** Cardiogenic shock, Heart failure related cardiogenic shock, Cardiogenic shock working group-modified society for cardiovascular angiography and interventions, Urine output, Mortality

## Abstract

**Background:**

The Cardiogenic Shock Working Group-modified Society for Cardiovascular Angiography and Interventions (CSWG-SCAI) has been validated in patients with cardiogenic shock (CS) related to heart failure (HF). Its prognostic value in patients with early-stage HF-CS has been scarcely investigated.

**Methods:**

In 208 patients with HF-CS, the relationship between the CSWG-SCAI stage at diagnosis, at 24 and 48 h, the maximum CSWG-SCAI stage, and in-hospital mortality were assessed. In addition, the added value of urine output (UO) to the CSWG-SCAI was evaluated.

**Results:**

At HF-CS diagnosis, stages A and B were most prevalent (33 % and 36 %), while stage C dominated at 24 h (51 %), 48 h (44 %) and maximum CSWG-SCAI (37 %). In total, 87 (42 %) patients died during hospitalization. At HF-CS diagnosis, lower stages (A/B) showed similar prognostic value compared to more severe stages (C/D/E) (p = 0.994). The CSWG-SCAI was associated with in-hospital mortality at 24 h (p = 0.005), 48 h (p = 0.005) and at maximum CSWG SCAI (p < 0.001). Stage deterioration after 24 h was associated with mortality (deteriorated vs. improved: p < 0.001). SCAI-UO showed modest additive predictive value at 48 h (AUC 0.67 vs. AUC 0.70; p = 0.015) and maximum SCAI compared to CSWG-SCAI (AUC 0.66 vs. AUC 0.69; p = 0.032).

**Conclusions:**

At the time of HF-CS diagnosis, the CSWG-SCAI classification failed to predict in-hospital mortality, suggesting that it may not adequately capture the severity of early-stage HF-CS. The CSWG-SCAI classification was associated with in-hospital mortality at 24 and 48 h and at maximum CSWG-SCAI. Incorporating UO into the CSWG-SCAI criteria minimally improved risk stratification.

## Graphical abstract

Graphical abstract depicting the study flow at the upper part of the figure. On the left, at the bottom of the figure, in-hospital mortality is stratified by CSWG-SCAI stage. On the right, at the bottom of the figure, the area under the receiver operating characteristic curve (AUC-ROC) for the CSWG-SCAI and SCAI-UO is compared for each timepoint.

**Figure:**
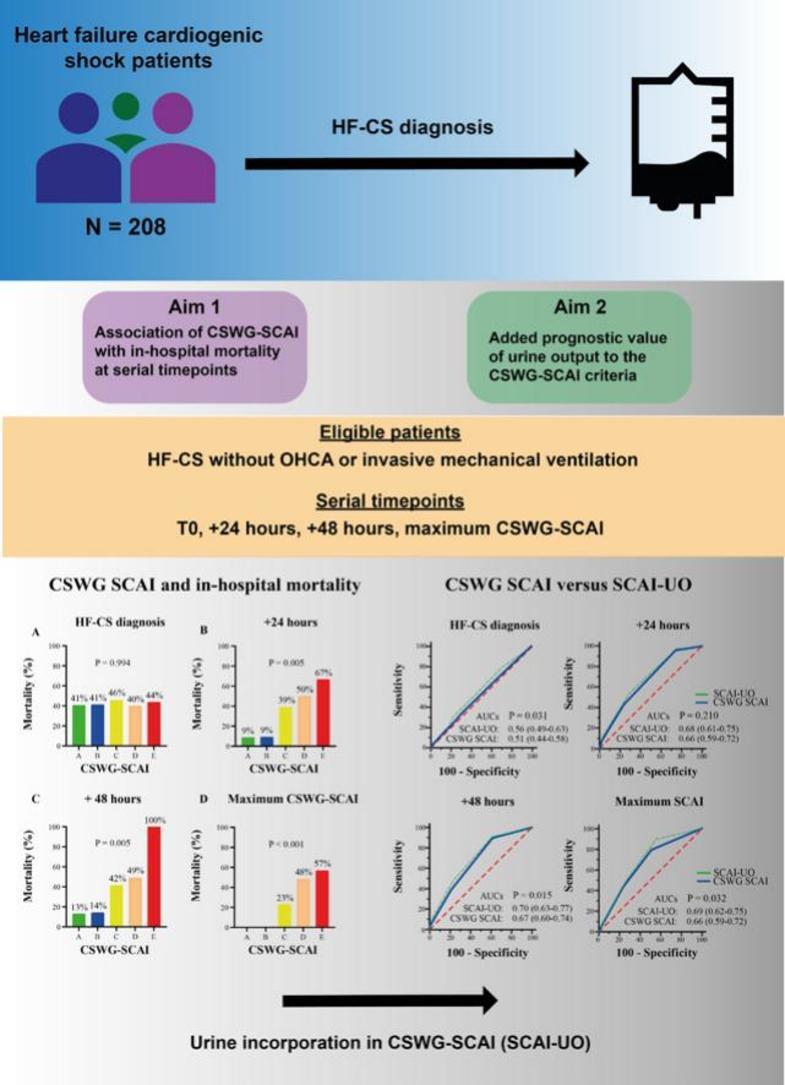
**Graphical abstract.** Graphical abstract depicting the study flow at the upper part of the figure. On the left, at the bottom of the figure, in-hospital mortality is stratified by CSWG-SCAI stage. On the right, at the bottom of the figure, the area under the receiver operating characteristic curve (AUC-ROC) for the CSWG-SCAI and SCAI-UO is compared for each timepoint.

## 1 Introduction

Cardiogenic shock (CS) is a critical condition in which impairment of cardiac function and/or structure leads to inadequate tissue perfusion. The two most important etiologies are CS related to acute myocardial infarction (AMI-CS) and acute heart failure (HF-CS) [[1], [2], [3]]. The majority of CS patients present with HF-CS, with a short-term reported mortality rate of 25–51 % [[4], [5], [6]]. An essential aspect of managing CS is the accurate assessment and classification of its severity during hospitalization. The Cardiogenic Shock Working Group (CSWG) adapted the original Society for Cardiovascular Angiography & Interventions (SCAI) classification to create the CSWG-SCAI, focusing on easily accessible clinical, laboratory, and treatment parameters for CS risk stratification. The CSWG-SCAI has been validated to predict short-term mortality in HF-CS [5,7], and a recent study suggested that serial assessment may further improve prognostic accuracy [8]. However, to date, evidence for the utility of the CSWG-SCAI criteria have primarily been investigated in advanced trajectories of HF-CS at the intensive care unit (ICU), consisting of mixed study populations, including CS patients after out-of-hospital cardiac arrest (OHCA) and/or treated with invasive mechanical ventilation (MV) [5]. Concerns have arisen regarding the precision of SCAI staging, particularly in earlier stages of CS, as well as its limited consideration for the heterogeneity of the disease [9]. Urine output (UO) is currently not included in the CSWG-SCAI classification, although it is widely used in clinical practice as a key parameter to detect early hypoperfusion and has been shown to predict mortality [[10], [11], [12]]. This study aims to: (1) evaluate the association at serial timepoints between the CSWG-SCAI stages and in-hospital mortality in HF-CS patients; (2) assess the added prognostic value of UO to the CSWG-SCAI criteria.

## 2 Methods

### 2.1 Study design

This is a retrospective, observational, multicenter study of patients hospitalized for acute HF between January 2015 and December 2021 at two university medical centers in the Netherlands. Inclusion criteria were: (1) age ≥ 18 years; (2) diagnosis of HF-CS. Exclusion criteria were: (1) CS due to acute myocardial infarction (AMI) (ST-segment elevation MI and non-ST-segment elevation MI); (2) post-cardiotomy shock; (3) invasive MV at time of HF-CS diagnosis; (4) OHCA; (5) left ventricular ejection fraction (LVEF) ≥ 45 % and (6) lost to follow-up. The diagnosis of HF-CS was established when the treating physician deemed CS directed therapy (vasoactive-inotropic therapy and/or MCS) necessary to treat acute HF. To capture an early-stage cohort of patients with HF-CS, we defined the time of HF-CS diagnosis, whether at hospital admission or later during hospitalization, as the starting point for analysis. To further refine this early-stage population, we excluded patients who had already undergone invasive MV at the time of HF-CS diagnosis and those with OHCA. Individuals with LVEF ≥ 45 % were excluded, as vasoactive-inotropic therapy was not considered appropriate for non-systolic heart failure. Patients were eligible for inclusion in the registry only if diagnosed with HF-CS during admission or during hospitalization.

A local institutional review board provided ethical approval for this study and waived the need for informed consent (2021.0734). This study was conducted in accordance with the Declaration of Helsinki and the guidelines of the institutional review board.

### 2.2 Data collection

Patient data, including demographics, patient history, vitals, laboratory*,* echocardiography, and therapeutic interventions were extracted from the electronic clinical records at five standardized timepoints during hospitalization: admission (within 6 h after admission), at HF-CS diagnosis but before the administration of vasoactive-inotropic support, at 24 and 48 h after HF-CS diagnosis and at the time of the maximum CSWG-SCAI stage. Patient characteristics were described using data at admission, while the time of HF-CS diagnosis was used as the starting point of analysis and for the subsequent 24 and 48 h analysis. Echocardiography was performed according to European guidelines [13] during hospital admission, or if unavailable, in the last six months prior to admission.

### 2.3 CSWG-SCAI

The SCAI stages were retrospectively assigned according to the CSWG-SCAI classification at each timepoint [7]. Cutoff values for each parameter used in the determination of the CSWG-SCAI stages are provided in Supplemental Table 1. In summary, the CS severity scoring system classifies patients into five stages (A-E) based on the severity of hypotension or hypoperfusion, treatment intensity with vasoactive-inotropic support and/or mechanical circulatory support (MCS), and the incidence of OHCA. In addition, we assessed the changes in CSWG-SCAI stage after 24 and 48 h of HF-CS diagnosis. The changes were categorized into three groups: stable, deteriorated, and improved. To assess these changes, the CSWG-SCAI was recalculated at HF-CS onset to account for the number of inotropes or vasopressors initiated, ensuring a more accurate evaluation of stage transitions.

### 2.4 SCAI-UO

A new SCAI was created that incorporated UO (SCAI-UO) in the CSWG-SCAI classification after initial statistical testing (Supplemental Table 2). UO below 0.5 mL/kg/hour (oliguria) was considered as hypoperfusion for stage B/C, while UO less than 0.3 mL/kg/hour was considered as stage D without hypotension and stage E with hypotension (SBP ≤ 90 mmHg, MAP ≤ 65 mmHg) [14]. Moreover, UO below 0.5 mL/kg/hour during vasoactive-inotropic support was considered as persistent hypoperfusion and classified as stage D.

### 2.5 Patient outcome

The primary outcome was in-hospital mortality, defined as all-cause mortality during hospitalization. Patients who had ongoing CS and were discharged in a palliative care setting were also classified as in-hospital deaths. The association between CSWG-SCAI stages and mortality, as well as the changes in CSWG-SCAI stages, were evaluated. Additionally, the prognostic performance of the CSWG-SCAI and SCAI-UO classifications were compared to determine their relative superiority.

### 2.6 Statistical analysis

Data are presented as mean ± (SD) or median (IQR) for continuous variables and as absolute numbers (%) for categorical variables. Continuous variables were compared using the independent *t*-test, Mann-Whitney U or Kruskal-Wallis test as appropriate. Categorical variables were compared using the chi-square or Fisher’s exact test. Logistic regression was used to model the association between in-hospital mortality and the CSWG-SCAI criteria, with CSWG-SCAI A as the reference class. Multivariate adjustment was performed for linear effects of continuously measured age (in years), hemoglobin (in mmol/L) and estimated glomerular filtration rate [>60 mL/min/1.73 m^2^] (eGFR) after univariate testing (Supplemental Table 2). Time-to-event analysis was performed at HF-CS diagnosis, at 24 h, and at 48 h with in-hospital mortality and discharge as competing risks. The cause-specific hazard models included CSWG-SCAI as a factor variable and assumed proportional effects. We conducted a multivariable regression analysis that incorporated the CSWG-SCAI stages and UO at each timepoint to assess the added value of UO in developing the SCAI-UO classification (Supplemental Table 2). The areas under the receiver operating characteristic curve (AUC-ROC) for the CSWG-SCAI and SCAI-UO to predict mortality were calculated and compared using the DeLong method. A two-sided p-value < 0.05 was considered statistically significant. Statistical analyses were performed using SPSS software (IBM SPSS Statistics 28.0, Chicago, IL), RStudio version 4.4.3 (R Foundation, Vienna, Austria) and MedCalc Statistical Software version 20.006 (MedCalc Software bv, Ostend, Belgium).

## 3 Results

### 3.1 Study population

**Fig. 1:**
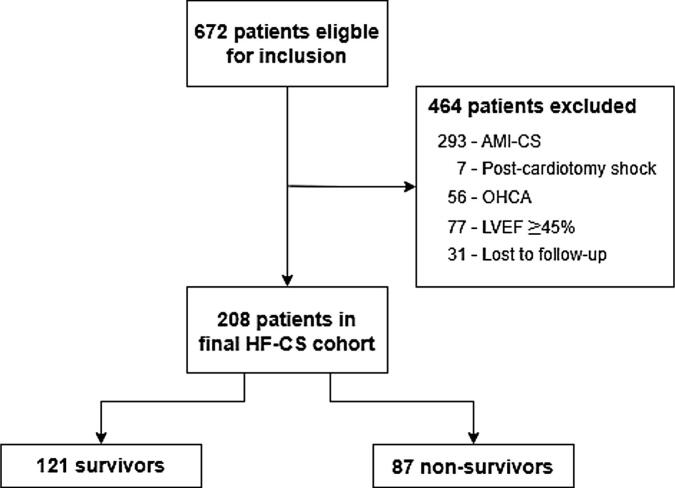
**Study flowchart** Study flowchart of included patients. Abbreviations: AMI-CS: acute myocardial infarction related cardiogenic shock, OHCA: Out-of-hospital cardiac arrest, MV: invasive mechanical ventilation, LVEF: Left ventricular ejection fraction, HF-CS: Heart failure related cardiogenic shock.

A total of 672 patients were eligible for inclusion in this study. Excluded patients were: 293 due to AMI-CS, 7 because of post-cardiotomy shock, 56 because of OHCA, 77 had a LVEF ≥ 45 % and 31 were lost to follow-up, resulting in a final cohort of 208 patients (Fig. 1). Patient characteristics are presented in Table 1. Median age was 73 (IQR: 64–80 years), and 62 (30 %) patients were female. A non-ischemic cardiomyopathy was observed in 112 (54 %) patients, 93 (45 %) had an ischemic cardiomyopathy and the diagnosis was undefined in 3 (1 %) patients. Thirty patients (14 %) were adjudicated as de novo HF-CS while the remainder 178 (86 %) were adjudicated as acute-on-chronic HF-CS. Echocardiography showed a poor (<30 %) left ventricular ejection fraction in 140 (69 %) patients, with 64 % of these studies performed during hospitalization and the remainder obtained within six months prior to admission.

**Table 1:** Patient characteristics between survivors and non-survivors at time of hospital admission.

	Total n = 208	Survivors n = 121	Non-survivors n = 87	p-Value
**Demographics**				
Age (years)	73 (64–80)	70 (60–77)	75 (68–81)	**0.002**
Female gender	62 (30)	36 (30)	26 (30)	0.983
BMI (kg/m^2^)	27 ± 5	27 ± 6	26 ± 5	0.275

**Medical history**				
Hypertension	124 (61)	71 (59)	53 (62)	0.646
Dyslipidemia	114 (56)	71 (59)	43 (51)	0.223
Diabetes mellitus	70 (34)	42 (35)	28 (33)	0.759
Smoking (current or former)	113 (55)	66 (55)	47 (55)	0.967
Prior PCI	57 (27)	38 (31)	19 (22)	0.127
Prior MI	64 (31)	41 (34)	23 (26)	0.251
Prior CABG	29 (14)	17 (14)	12 (14)	0.958
Pacemaker	47 (23)	26 (22)	21 (24)	0.652
ICD	57 (27)	33 (27)	24 (28)	0.960
Peripheral arterial disease	28 (14)	16 (13)	12 (14)	0.872
Prior Stroke/TIA	35 (17)	22 (18)	13 (15)	0.562
Obstructive lung disease	40 (19)	24 (20)	16 (19)	0.825
Active cancer	23 (11)	11 (9)	12 (14)	0.273
CKD (KDIGO)	63 (30)	34 (28)	29 (34)	0.386

**Clinical presentation**				
De Novo HF	30 (14)	19 (16)	11 (13)	0.536
Etiology				0.221
Ischemic	93 (45)	60 (50)	33 (38)	
Non-Ischemic	112 (54)	59 (49)	53 (61)	
Unknown	3 (1)	2 (2)	1 (1)	
ECG				
Rhythm				0.255
Sinus	87 (42)	55 (46)	32 (37)	
SVT	54 (26)	30 (25)	24 (28)	
Pacemaker	55 (26)	29 (24)	26 (30)	
Other	12 (6)	7 (6)	5 (6)	
Left Bundle branch block	82 (39)	47 (39)	35 (40)	0.840
AKI on admission (KDIGO)	102 (55)	56 (51)	46 (60)	0.233

**Heart failure medication, N (%)**				
β-Blocker	133 (66)	78 (65)	55 (66)	0.852
ACE inhibitor/ARB	94 (46)	59 (49)	35 (42)	0.326
ARNI	17 (8)	9 (8)	8 (10)	0.589
MRA	112 (55)	69 (58)	43 (52)	0.423
SGLT-2I	9 (4)	6 (5)	3 (4)	0.637

**Echocardiography**				
LVEF (%)				0.941
30–44	63 (31)	37 (31)	26 (31)	
<30	140 (69)	83 (69)	57 (69)	
Severe RV dysfunction, N (%)	36 (19)	25 (22)	11 (15)	0.213
Severe valvular heart disease, N (%)				
Aortic stenosis	8 (4)	1 (1)	7 (10)	**0.009**
Mitral valve regurgitation	31 (17)	17 (15)	14 (19)	0.547
Tricuspid valve regurgitation	37 (21)	21 (19)	16 (23)	0.542
LVEDD (cm)	6.2 ± 1.0	6.2 ± 1.0	6.1 ± 1.2	0.665

**Vitals & laboratory values**				
Heart rate, bpm	88 ± 28	93 ± 30	81 ± 23	**0.004**
Systolic blood pressure, mmHg	107 ± 27	112 ± 28	101 ± 24	**0.003**
Mean arterial pressure, mmHg	80 ± 19	84 ± 19	74 ± 16	**<0.001**
Hemoglobin, mmol/L	8.1 ± 5.1	8.6 ± 6.7	7.4 ± 1.3	0.101
Platelet count, x10^9^/L	217 ± 79	224 ± 79	207 ± 78	0.184
ALAT, U/L	90 ± 175	102 ± 207	74 ± 117	0.406
NT-proBNP, ng/L	11,143 (4789–27450)	8815 (3870–18466)	16,905 (8118–30812)	**<0.001**
Creatinine, µmol/L	197 ± 125	171 ± 96	233 ± 149	**<0.001**
eGFR, CKD-EPI	39 ± 31	46 ± 37	30 ± 17	**<0.001**
BUN, mmol/L	19.3 ± 12.4	15.7 ± 11.4	24.1 ± 12.3	**<0.001**
pH	7.35 ± 0.13	7.34 ± 0.14	7.37 ± 0.11	0.293
Lactate, mmol/L	3.5 ± 3.3	3.5 ± 2.3	3.5 ± 4.6	0.973

**Admission CSWG-SCAI**				
CSWG-SCAI				0.613
A	116 (56)	67 (55)	49 (56)	
B	56 (27)	34 (28)	22 (25)	
C	11 (5)	4 (3)	7 (8)	
D	6 (3)	4 (3)	2 (2)	
E	19 (9)	12 (10)	7 (8)	

Values are presented as mean ± SD, or absolute numbers (%).

Abbreviations: BMI: Body mass index, PCI: Percutaneous coronary intervention, MI: myocardial infarction, CABG: Coronary artery bypass graft surgery, ICD: Implantable cardioverter-defibrillator, TIA: Transient ischemic attack, CKD: Chronic kidney disease, HF: Heart failure, ECG: Electrocardiogram, SVT: Supra-ventricular tachycardia, AKI: Acute kidney injury, ACE: Angiotensin-converting enzyme, angiotensin receptor blocker, ARNI: Angiotensin receptor/neprilysin inhibitor, MRA: Mineralocorticoid receptor antagonist, SGLT-2i: Sodium-glucose cotransporter-2 inhibitor, LVEF: Left ventricular ejection fraction, RV: Right ventricle, LVEDD: Left ventricular end diastolic diameter, ALAT: alanine aminotransferase, ASAT: aspartate aminotransferase, eGFR: Estimated Glomerular Filtration Rate, BUN: Blood urea nitrogen, KDIGO: Kidney Disease: Improving Global Outcomes.

At hospital admission, heart rate, blood pressure, and renal function significantly differed between survivors and non-survivors. At this timepoint, the CSWG-SCAI distribution was as follows: stage A: 56 % (n = 116), stage B: 27 % (n = 56), stage C: 5 % (n = 11), stage D: 3 % (n = 6), and stage E: 9 % (n = 19). CSWG-SCAI at admission did not differ between survivors and non-survivors (p = 0.613). The median time until HF-CS onset was 48 h (5–146 h) after hospital admission, with 62 (30 %) patients presenting with HF-CS at the time of hospital admission.

### 3.2 Invasive therapies and monitoring

Table 2 shows the invasive therapies and monitoring. Dobutamine (45 %) and norepinephrine (42 %) were the most commonly used inotropic/vasoactive drugs. The majority of the patients received one inotrope or vasoactive (78 %). MCS was initiated in 1 (1 %) patient, and renal replacement therapy in 6 (3 %) patients. In total, 23 (11 %) patients were admitted to the ICU and 10 (5 %) patients were treated with invasive MV after the onset of HF-CS. The median hospitalization duration was 15 days (IQR: 7–24 days). The duration of hospitalization stratified by CSWG–SCAI stage at each time point is presented in Supplemental Table 3. At each timepoint, the duration of hospitalization differed significantly across the corresponding CSWG-SCAI stages.

**Table 2:** Invasive therapies and monitoring between survivors and non-survivors.

	Total n = 208	Survivors n = 121	Non-survivors n = 87	p-Value
**Invasive therapy**				
Vasoactives/inotropes at admission, N (%)	62 (30)	39 (32)	23 (26)	0.367
Time between hospitalization and first administration (hours)	48 (5–146)	36 (5–132)	50 (6–182)	0.273
Type of drug				
Norepinephrine	87 (42)	50 (41)	37 (43)	0.862
Enoximone	35 (17)	23 (19)	12 (14)	0.321
Milrinone	11 (5)	7 (6)	4 (5)	0.765
Dobutamine	93 (45)	52 (43)	41 (47)	0.553
Dopamine	33 (16)	18 (15)	15 (17)	0.645
Mechanical circulatory support	1 (1)	0 (0)	1 (1)	0.418
Intensive care admission	23 (11)	12 (10)	11 (13)	0.536
Mechanical ventilation	10 (5)	3 (3)	7 (8)	0.099
PAC use	8 (4)	5 (4)	3 (3)	>0.999
RRT	6 (3)	1 (1)	5 (6)	0.084
Valve surgery or intervention	7 (3)	5 (4)	2 (2)	0.702
**Advanced therapy**				
LVAD	2 (1)	2 (2)	0 (0)	0.511
HTx	0 (0)	0 (0)	0 (0)	−

Values are presented as mean ± SD, median (IQR) or absolute numbers (%).

Abbreviations: PAC: Pulmonary artery cathether, RRT: Renal replacement therapy, LVAD: Left ventricular assist device, HTx: Heart transplantation.

### 3.3 CSWG-SCAI trajectory

**Fig. 2:**
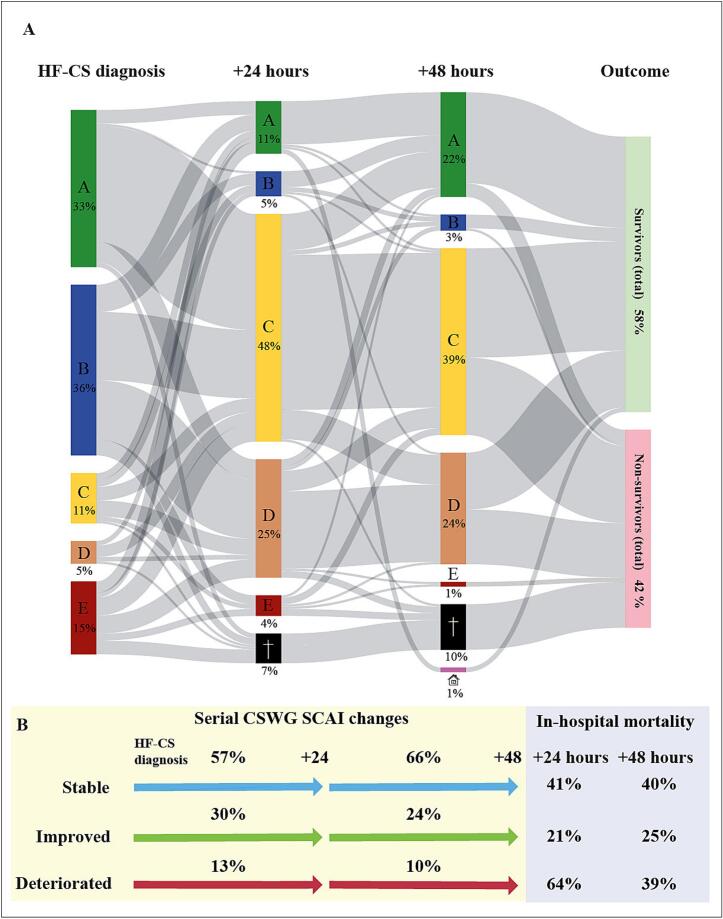
**CSWG-SCAI trajectory during vasoactive-inotropic support (A)** CSWG-SCAI stage distribution for each timepoint. **(B)** Serial changes in CSWG-SCAI stage with associated mortality rates for each timepoint. Abbreviations: CSWG-SCAI: Cardiogenic Shock Working Group modified Society for Cardiovascular Angiography & Interventions.

Fig. 2a presents the CSWG-SCAI trajectory during the first 48 h after HF-CS diagnosis. At HF-CS onset, but before the administration of vasoactive-inotropic support, the CSWG-SCAI stage distribution was: A: 33 %, B: 36 %, C: 11 %, D: 5 %, and E: 15 %. Stage determination was in 50 % of the patients based on hypotension, hypoperfusion in 27 %, and a combination of both in 23 % (Supplemental Fig. 1). Lactate was the predominant factor related to hypoperfusion (61 %) at this timepoint. (Supplemental Fig. 2).

The classification shifted at 24 h: A: 11 %, B: 5 %, C: 48 %, D: 25 %, and E: 4 %. At 48 h, stage A increased to 22 %, while C (39 %) and D (24 %) remained the most prevalent. Determination at both 24 and 48 h was primarily determined by the number of vasoactive/inotropic agents, 53 % and 61 % respectively.

The maximum CSWG-SCAI stages were C: 37 %, D: 31 % and E: 32 %. The median time to the maximum CSWG-SCAI stage was 3 days (IQR: 0–8 days). The number of vasoactive-inotropic agents with concomitant (persistent) hypotension and/or hypoperfusion was the most important stage determinant at maximum CSWG-SCAI (35 %), followed by the number of vasoactives/inotropes (32 %).

Over the 48-hour trajectory, mean lactate levels decreased following HF-CS diagnosis, whereas mean ALAT levels continued to rise throughout the first 48 h. By the end of the 48-hour period, vasoactive-inotropic support had been discontinued in 29 % of the patients (Table 3). The prognostic relevance of individual CSWG-SCAI components is detailed in Supplemental Table 4. At the time of HF-CS diagnosis, none of the individual variables demonstrated prognostic significance. At 24 and 48 h, and maximum CSWG-SCAI, the number of vasoactive-inotropes, along with persistent hypotension or hypoperfusion despite therapy, emerged as the strongest predictors for in-hospital mortality.

**Table 3:** Clinical and laboratory parameters for each timepoint during inotropic/vasoactive treatment and maximum CSWG-SCAI.

	HF-diagnosis n = 208	+24 h n = 195	+48 h n = 186	Maximum CSWG-SCAI n = 208
**CSWG-SCAI variables**				
Systolic blood pressure, mmHg	96 ± 25	104 ± 19	110 ± 19	96 ± 27
Mean arterial pressure, mmHg	71 ± 18	76 ± 13	79 ± 12	71 ± 19
pH	7.37 ± 0.13	7.41 ± 0.09	7.40 ± 0.10	7.34 ± 0.15
Lactate, mmol/L	3.3 ± ± 2.2	2.2 ± 1.5	2.4 ± 2.0	4.1 ± 3.6
ALAT, U/L	212 ± 388	562 ± 857	662 ± 724	310 ± 656

Number of drugs				
0	NA	36 (19)	53 (29)	21 (10)
1	162 (78)	115 (59)	97 (52)	120 (58)
2	46 (22)	42 (22)	36 (19)	57 (27)
3+	0 (0)	2 (1)	0 (0)	10 (6)
Number of MCS	0	0 (0)	0 (0)	1 (0)
1 drug or device with persistent hypotension hypoperfusion	NA	25 (13)	25 (13)	20 (10)
Urine output, mL	1174 ± 603	2021 ± 1452	2182 ± 1335	1098 ± 674

Values are presented as mean ± SD, median (IQR) or absolute numbers (%).Abbreviations: CSWG-SCAI: Cardiogenic Shock Working Group modified Society for Cardiovascular Angiography & Interventions.

### 3.4 CSWG-SCAI and in-hospital outcomes

**Fig. 3:**
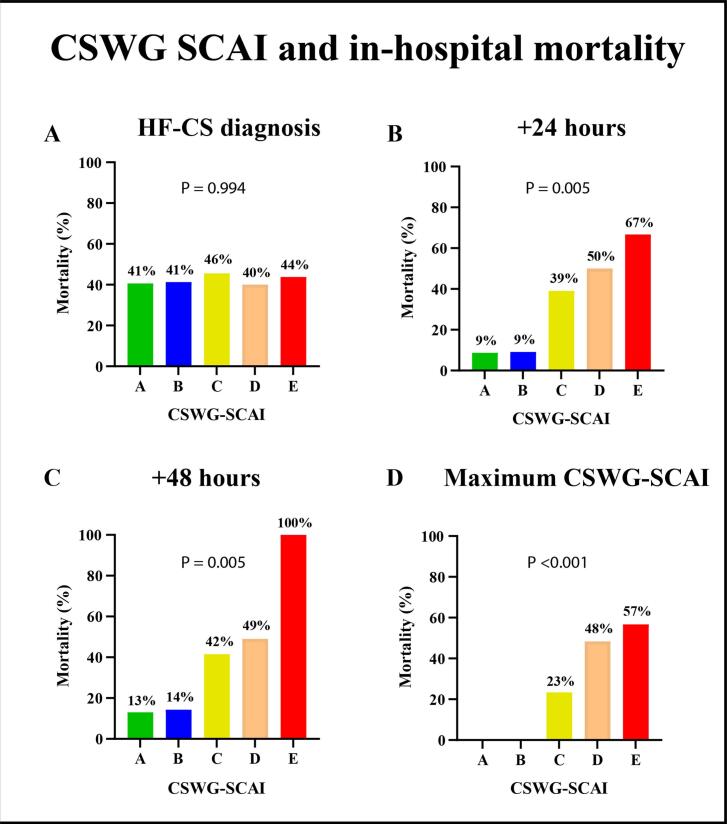
**CSWG-SCAI and in-hospital mortality.** In-hospital mortality stratified by CSWG-SCAI stage **(A)** At time of HF-CS diagnosis **(B)** 24 h after HF-CS diagnosis **(C)** 48 h after HF-CS diagnosis **(D)** Maximum CSWG-SCAI.

A total of 87 (42 %) patients died during hospitalization and one (1 %) patient received a left ventricular assist device (Table 2). Mortality rates after 24 and 48 h were 7 % and 10 %, respectively. Fig. 3 depicts the in-hospital mortality stratified by CSWG-SCAI stage. At HF-CS onset, no relationship between CSWG-SCAI-stage and in-hospital mortality was observed (p = 0.994). At 24 h the CSWG-SCAI was significantly associated with in-hospital mortality (p = 0.005). Compared with stage A, the odds ratios (OR) were: B: OR 1.1 (95 % CI:0.09–13.0, p = 0.970), C: OR 6.7 (95 % CI: 1.5–30.2, p = 0.013), D: OR 10.5 (95 % CI: 2.2–49.4, p = 0.003) and E: OR 21 (95 % CI: 2.8–156.1, p = 0.003). After 48 h, the CSWG-SCAI remained significantly associated with in-hospital mortality (p = 0.005). Compared to stage A, the OR’s for in-hospital mortality were: B: OR 1.1 (95 % CI: 0.1–10.9, p = 0.928), C: OR 4.7 (95 % CI: 1.8–12.4, p = 0.002), D: OR 6.4 (95 % CI: 2.3–7.8, p < 0.001) and in E all patients died. The maximum CSWG-SCAI stage showed an incremental risk for in-hospital mortality for each stage compared to stage C (p < 0.001): D: OR 3.1 (95 % CI: 1.5–6.3, p = 0.002) and E: OR 4.3 (95 % CI: 2.1–8.8, p < 0.001). The CSWG-SCAI classification remained an independent predictor of in-hospital mortality after multivariate adjustment after 24 h (p = 0.039), 48 h (p = 0.024), and maximum CSWG-SCAI (p < 0.001). Supplemental Fig. 3 presents the time-dependent survival analyses for patients with HF-CS at diagnosis, at 24 and 48 h. CSWG–SCAI stage at the time of HF-CS diagnosis was not significantly associated with in-hospital mortality. However, when reassessed at 24 and 48 h, higher CSWG–SCAI stages were progressively associated with increased cumulative probability of in-hospital death.

### 3.5 CSWG-SCAI stage serial changes

The association between the serial changes in CSWG-SCAI stages and in-hospital mortality is presented in Fig. 2b. After 24 h, 57 % of the patients remained stable, 30 % improved and 13 % deteriorated. From 24 h to 48 h, 66 % of the patients remained stable, 24 % improved and 10 % deteriorated in CSWG-SCAI stage. The highest mortality was found in patients who deteriorated within the first 24 h (64 %). Patients who improved at 24 h had the lowest mortality rate (21 %). After 24 h a significant association between the change in CSWG-SCAI stage and mortality was found for all comparisons: Stable vs. improved: OR 0.4 (95 % CI: 0.18–0.78, p = 0.009), stable vs. deteriorated: OR 2.6 (95 % CI: 1.0–6.3, p = 0.041) and deteriorated vs. improved: OR 0.1 (95 % CI: 0.1–0.4, p < 0.001). After multivariate adjustment, stable vs. deteriorated (p = 0.018) and deteriorated vs. improved (p = 0.008) remained significantly associated with in-hospital mortality. Serial changes assessed between 24 and 48 h did not reach statistical significance in any of the pairwise comparisons.

### 3.6 SCAI-UO

**Fig. 4:**
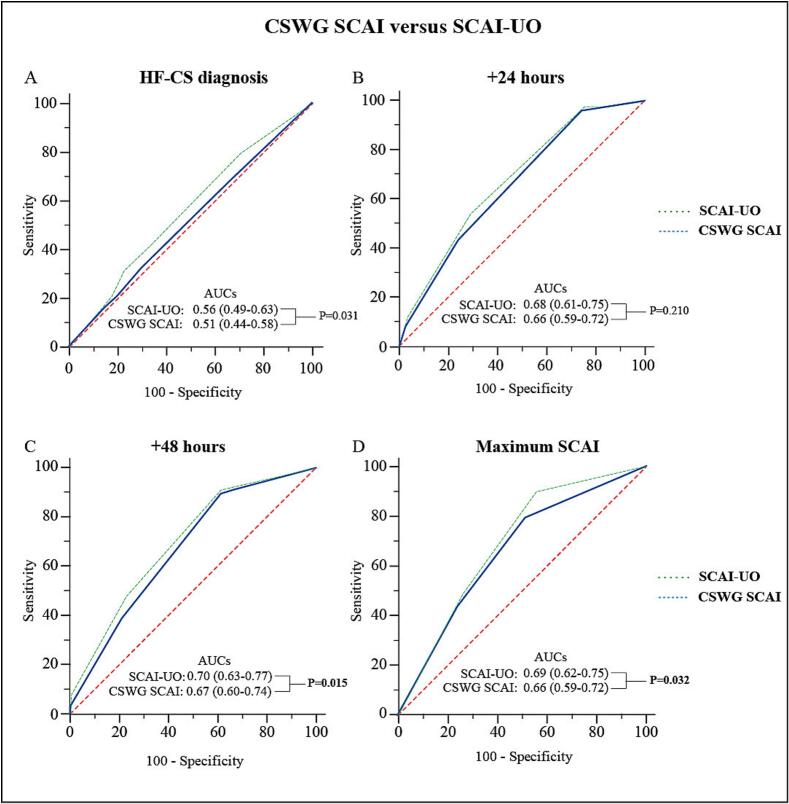
**Comparison of AUC-ROC of the CSWG-SCAI and SCAI-UO for each timepoint. (A)** At the time of HF-CS diagnosis **(B)** 24 h after HF-CS diagnosis **(C)** 48 h after HF-CS diagnosis **(D)** Maximum CSWG-SCAI. Abbreviations: SCAI-UO: Society for Cardiovascular Angiography & Interventions with urine output incorporation.

Availability of UO varied across the timepoints (Supplemental Table 5). At all intervals, UO was significantly associated with in-hospital mortality, also after adjustment for the corresponding CSWG-SCAI classification (Supplemental Table 2). In our cohort, 28 (14 %) patients experienced oliguria at HF-CS diagnosis, 27 (13 %) patients at 24 h, 18 (9 %) patients at 48 h, and 47 (23 %) patients at maximum SCAI-UO. Table 4 highlights the differences in stage distribution between the CSWG-SCAI and SCAI-UO. The percentage of patients that modified the SCAI stage when classified based on SCAI-UO ranged from 8 % to 11 %. Most shifts were observed at HF-CS onset (11 %). After 24, 48 h and at maximum SCAI-UO, SCAI-UO remained a significant predictor of in-hospital mortality, also after multivariate adjustment (p = 0.046, p = 0.016, and p < 0.001, respectively). Fig. 4 shows the comparison of the ROC-AUCs at all timepoints. A significant difference between the ROC-AUCs of CSWG-SCAI and SCAI-UO was observed at HF-CS onset (p = 0.031). However, when analyzed independently, the ROC-AUC of SCAI-UO did not reach statistical significance (p = 0.116). Pairwise comparison at 24 h was not significant (p = 0.210). A statistically significant, but very small, difference was found between the two curves at 48 h (p = 0.015) and maximum SCAI (p = 0.032). The AUC for the CSWG-SCAI at 48 h was 0.67 (95 % CI: 0.60–0.74), while the AUC for SCAI-UO was 0.70 (95 % CI: 0.63–0.77). For maximum CSWG-SCAI the AUC was 0.66 (95 % CI: 0.59–0.72), compared to 0.69 (95 % CI: 0.62–0.75) for maximum SCAI-UO.

**Table 4:** Comparison of SCAI distribution between CSWG-SCAI and SCAI-UO.

	HF-CS diagnosis n = 208		+24 h n = 195		+48 h*** n = 186		Maximum SCAI n = 208	
	CSWG-SCAI	SCAI-UO	CSWG-SCAI	SCAI-UO	CSWG-SCAI	SCAI-UO	CSWG-SCAI	SCAI-UO
A	69 (33)	54 (26)	23 (11)	23 (11)	46 (22)	44 (21)	NA	NA
B	75 (36)	78 (37)	11 (5)	9 (4)	7 (3)	8 (4)	NA	NA
OR	1.0	1.5	1.1	0.0	1.1	1.1	NA	NA
C	22 (11)	22 (11)	100 (48)	88 (42)	82 (39)	75 (36)	77 (37)	63 (30)
OR	1.2	1.4	6.7	6.0	4.7	4.9	**	**
D	10 (5)	15 (7)	52 (25)	62 (30)	49 (24)	54 (26)	64 (31)	72 (35)
OR	1.0	3.0	10.5	10.5	6.4	7.8	3.1	6.0
**E**	32 (15)	39 (19)	9 (4)	13 (6)	2 (1)	5 (2)	67 (32)	73 (35)
OR	1.1	1.7	21	23.6	*	*	4.3	8.1
Death	NA	NA	13 (7 %)		20 (10 %)		NA	NA
Changed	23 (11)		17 (9)		14 (8)		19 (9)	
p	0.994	0.428	0.005	0.008	0.005	0.003	<0.001	<0.001

Values are presented as absolute numbers (%).

*All patients died.

** Used as reference variable.

*** At 48 h two patients (1%) were discharged.

Abbreviations: SCAI-UO: Society for Cardiovascular Angiography & Interventions with urine output incorporated.

## 4 Discussion

This study investigates the applicability of the CSWG-SCAI classification in patients with HF-CS. The key findings of this study are: (1) At the time of HF-CS diagnosis, the CSWG-SCAI classification fails to predict in-hospital mortality; (2) At this timepoint, one third of the patients were deemed hemodynamically stable according to the CSWG-SCAI staging system (SCAI A); (3) The CSWG-SCAI classification is significantly associated with in-hospital mortality at 24 and 48 h after HF-CS diagnosis, as well as at the maximum CSWG-SCAI; (3) deterioration in CSWG-SCAI stage at 24 h is an important predictor for in-hospital mortality; and (4) SCAI-UO showed only a minimal improvement in predictive value for in-hospital mortality compared to the CSWG-SCAI classification at 48 h and the maximum SCAI-UO stage.

### 4.1 CSWG-SCAI classification

Interestingly, in contrast to previous research on HF-CS, the CSWG-SCAI classification at time of HF-CS diagnosis did not show a relationship with in-hospital mortality [[5], [7], [15]]. In our study, the majority of patients had not yet exhibited clinical signs of classic shock (CSWG-SCAI A-B) at the time of hospital admission (83 %) or at HF-CS diagnosis (69 %). The absence of a significant correlation at this timepoint is in part attributable to the high mortality rate observed for CSWG-SCAI stage A. There are multiple potential explanations. First, the clinicians’ decision to initiate therapy at the time of HF-CS diagnosis may have been appropriate, meaning that the patient was actually more critical ill than was captured by the current CSWG-SCAI criteria. This may suggest that the CSWG-SCAI system does not fully encompass the complexity of HF-CS patients in early shock stages (CSWG-SCAI A/B). Recently, it was demonstrated that many AHF patients admitted to the CICU exhibited isolated low cardiac output without accompanying hypotension or hypoperfusion, as assessed by laboratory results [16]. Classical indicators of hypoperfusion may not yet be evident, making it essential to rely on a broader – yet to be determined – set of diagnostic markers to detect HF-CS [17,18]. Second, physicians might have initiated vasoactive-inotropic support inappropriately, potentially contributing to adverse effects on patient survival. Both the American College of Cardiology /American Heart Association and European Society of Cardiology advocate against the use vasoactive-inotropic support in the absence of hypotension and/or hypoperfusion, as previous studies have demonstrated an increased mortality risk [19,20]. Despite these recommendations, vasopressor-inotropic therapy remains prevalent in this patient category [20,21]. Therefore, the possibility of overtreatment or misapplication of staging in patients who may not have met formal criteria for cardiogenic shock cannot be excluded. Although misclassification may have occurred in some cases, the observed high mortality among patients classified as CSWG-SCAI Stage A suggests that clinical judgment was, in many instances, appropriate. It is unlikely that mortality attributable to inotropic therapy alone fully accounts for this finding. Furthermore, the CSWG-SCAI is not a diagnostic tool, but rather a severity staging instrument. The CSWG-SCAI classification does not incorporate physical examination findings, renal function, or other relevant clinical parameters. Appropriate initiation of therapy may occur outside the scope of the classification framework. Third, our study population shows important different characteristics compared to other HF-CS cohorts. Our patients did not have a primary indication for invasive MV or OHCA, were significantly older and the majority (86 %) presented with acute-on-chronic HF-CS, the latter two known to be independent predictors of mortality [6,22]. Additionally, this may have resulted in more restricted treatment policies, fewer escalations to the ICU and limited use of MCS compared to study cohorts in existing literature. Given the potential limitations in prognostication using the initial CSWG-SCAI stages in this cohort, further exploration of easily accessible parameters is needed. These parameters could enhance accurate staging and risk stratification, leading to optimal (timing of) treatment, and potentially improved prognosis.

Serial CSWG-SCAI assessments at 24 and 48 h improved prognostic accuracy for in-hospital mortality. [8,23]. The number of vasoactive-inotropes and persistent hypotension or hypoperfusion were the strongest predictors within the CSWG-SCAI classification. Moreover, deterioration in CSWG-SCAI stage at 24 h was a strong predictor for mortality. Changes in CSWG-SCAI stage beyond 24 h did not further improve predictive accuracy. Our findings align with recent studies demonstrating that re-staging after 24 h enhanced prognostic accuracy. Similarly, it was recently demonstrated that changes in CSWG-SCAI stage occurred most frequently within the first 24 h, a period referred to as the ‘golden day of shock,’ beyond which further stage changes did not contribute to additional prognostic information [8].

A strong association between the maximum CSWG-SCAI and in-hospital mortality was found in our study, which is in line with previous findings [5,7]. Moreover, the median time to reach the maximum CSWG-SCAI stage was three days, underscoring the critical importance of early stabilization to halt CSWG-SCAI progression. However, the clinical implications of these findings remain uncertain, particularly given that maximum CSWG-SCAI stage is assessed retrospectively and without a standardized time point**.** Except for patients classified as CSWG-SCAI stage E, it is currently not possible to predict when a patient will reach its maximum CSWG-SCAI stage. Furthermore, it is arbitrary when the most severe SCAI stage is assessed, as values tend to worsen the closer they are measured to the time of death, with no clear guidance from previous literature [5,7].

For real-world practice, the CSWG-SCAI classification assessed at shock onset (T0) has limited prognostic accuracy, underscoring the continued importance of clinical assessment, invasive hemodynamics, and possibly kidney function in risk stratification. However, serial reassessment of CSWG-SCAI stage at 24 h and beyond provides a valuable tool for guiding treatment decisions and may improve outcomes through standardized escalation or de-escalation of therapy.

Interestingly, a lower heart rate at hospital admission was observed in the non-surviving group. Beta-blocker use was comparable between the groups, although no information about dosage was available. However, only 30 % of patients presented with HF-CS at admission, while the remaining 70 % developed HF-CS during hospitalization and were not yet critically ill at presentation. Notably, patients with HF-CS at admission had lower mortality (37 %) compared to those with later onset (44 %), suggesting that a higher heart rate – serving as a surrogate for early, compensatory HF-CS – may be associated with improved survival, especially in patient with acute-on-chronic HF-CS.

### 4.2 The added value of incorporating UO in the CSWG SCAI criteria

In an attempt to optimize the CSWG-SCAI classification, we investigated the added prognostic value of UO to the criteria. When evaluated as a continuous variable, UO consistently emerged as an independent predictor of in-hospital mortality across all time points. However, when incorporated into the CSWG-SCAI classification using predefined thresholds from the existing literature, SCAI-UO demonstrated only a marginal prognostic advantage – limited to the 48-hour and maximum SCAI-UO assessments – raising serious questions about the clinical significance of this modest difference. There are mixed findings in literature regarding UO, and only based on mixed CS cohorts. Some studies showed that UO < 0.5 mL/kg/h within the first 24 h after ICU admission only predicted mortality in SCAI stages D/E [11]. Whereas others found that only UO < 0.3 mL/kg/h predicted mortality in all SCAI stages [24].

Diuretic management was not incorporated into the SCAI-UO, in keeping with the low-complexity design of the original CSWG-SCAI framework. However, the substantial variability in diuretic protocols in HF-CS may influence UO and could, in part, account for the limited prognostic value observed, as increased urine output in some patients likely reflected pharmacologic intervention rather than intrinsic clinical improvement. It is important to note that in our study at the time of HF-CS diagnosis, the CSWG-SCAI classification itself lacked any prognostic accuracy. Given this limitation, adding a single parameter like UO to the criteria is unlikely to meaningfully improve a staging system that has already proven to be inaccurate at this timepoint. These considerations warrant further investigation for inclusion of UO in future SCAI classifications.

### 4.3 Limitations

The retrospective design of the study may have introduced inherent biases in data collection, patient selection, and the context of clinical decision-making, while the limited sample size reduces the statistical power to detect meaningful association. Missing data may have influenced CSWG-SCAI staging, as abnormal values could shift patients to a higher stage. However, the missing variables in this study primarily reflect clinical judgment not to obtain certain laboratory tests, rather than random data omission. As this study included only patients with a LVEF < 45 %, no conclusions can be drawn regarding patients with preserved or mildly reduced LV3EF. Because some patients died during the first 48 h after onset of HF-CS, this might have introduced survival bias at later timepoints. Maximum CSWG-SCAI stage may have been prone to bias, as measurements close to death may have led to worse abnormalities and may have reflected the timing of measurement rather than the severity of illness itself. Diuretic management was not protocolized and may have introduced bias regarding UO/diuretic resistance. The comparison of AUCs between CSWG-SCAI and SCAI-UO was performed using the DeLong test on the same dataset used for model development, without bootstrapping or external validation, and is therefore subject to potential overfitting. Finally, our data is from 2015 to 2021 and may reflect outdated CS management with respect to the choice of inotropic support and heart failure medical therapies.

## 5 Conclusions

In HF-CS patients at the time of HF-CS diagnosis, the CSWG-SCAI classification fails to predict in-hospital mortality, suggesting that it may not adequately capture the severity of early-stage HF-CS. The CSWG-SCAI classification is associated with in-hospital mortality at 24 and 48 h after onset of HF-CS, as well as at the maximum CSWG-SCAI stage. Deterioration in CSWG-SCAI stage after 24 h emerged as a strong predictor of in-hospital mortality. Incorporating UO into the CSWG-SCAI classification slightly improved risk stratification in HF-CS patients.

## Data availability

The data underlying this article will be shared on reasonable request to the corresponding author.

## Author statement

This author takes responsibility for all aspects of the reliability and freedom from bias of the data presented and their discussed interpretation.

## Disclosures

Nothing to Disclose | No Sources of funding.

## CRediT authorship contribution statement

**Tijmen H. Ris:** Writing – review & editing, Writing – original draft, Visualization, Methodology, Investigation, Formal analysis, Data curation, Conceptualization. **Morsal Atazadah:** Visualization, Investigation. **Roel Hoek:** Writing – review & editing. **Jeroen Hoogland:** Writing – review & editing, Methodology. **Tim Balthazar:** Writing – review & editing. **Federico Pappalardo:** Writing – review & editing. **Paul Knaapen:** Writing – review & editing. **Mariëlle C. van de Veerdonk:** Writing – review & editing, Supervision, Methodology, Conceptualization. **Alexander Nap:** Writing – review & editing, Visualization, Supervision, Investigation, Conceptualization.

## Declaration of competing interest

The authors declare that they have no known competing financial interests or personal relationships that could have appeared to influence the work reported in this paper.

## Appendix A Supplementary data

Supplementary Fig. 1 **CSWG-SCAI determinants.** Parameters used for CSWG-SCAI determination at each timepoint. Abbreviations: #: Number of, H/H: Hypotension/Hypoperfusion.

Supplementary Fig. 2 **Hypoperfusion determinants for each timepoint.** Abbreviations: CSWG-SCAI: Cardiogenic Shock Working Group modified Society for Cardiovascular Angiography & Interventions

Supplementary Fig. 3 **Time-to-event analysis CSWG-SCAI and in-hospital mortality** **(A)** HF-CS diagnosis **(B)** at 24 hours **(C)** at 48 hours
