# Supplementary figures and images for: Predictive value of the cardiogenic shock working group-modified SCAI criteria in early-stage heart failure-related cardiogenic shock
Source: Int J Cardiol Heart Vasc. 2025 Aug 26;60:101776. doi: 10.1016/j.ijcha.2025.101776 (PMC12398876; doi:10.1016/j.ijcha.2025.101776)

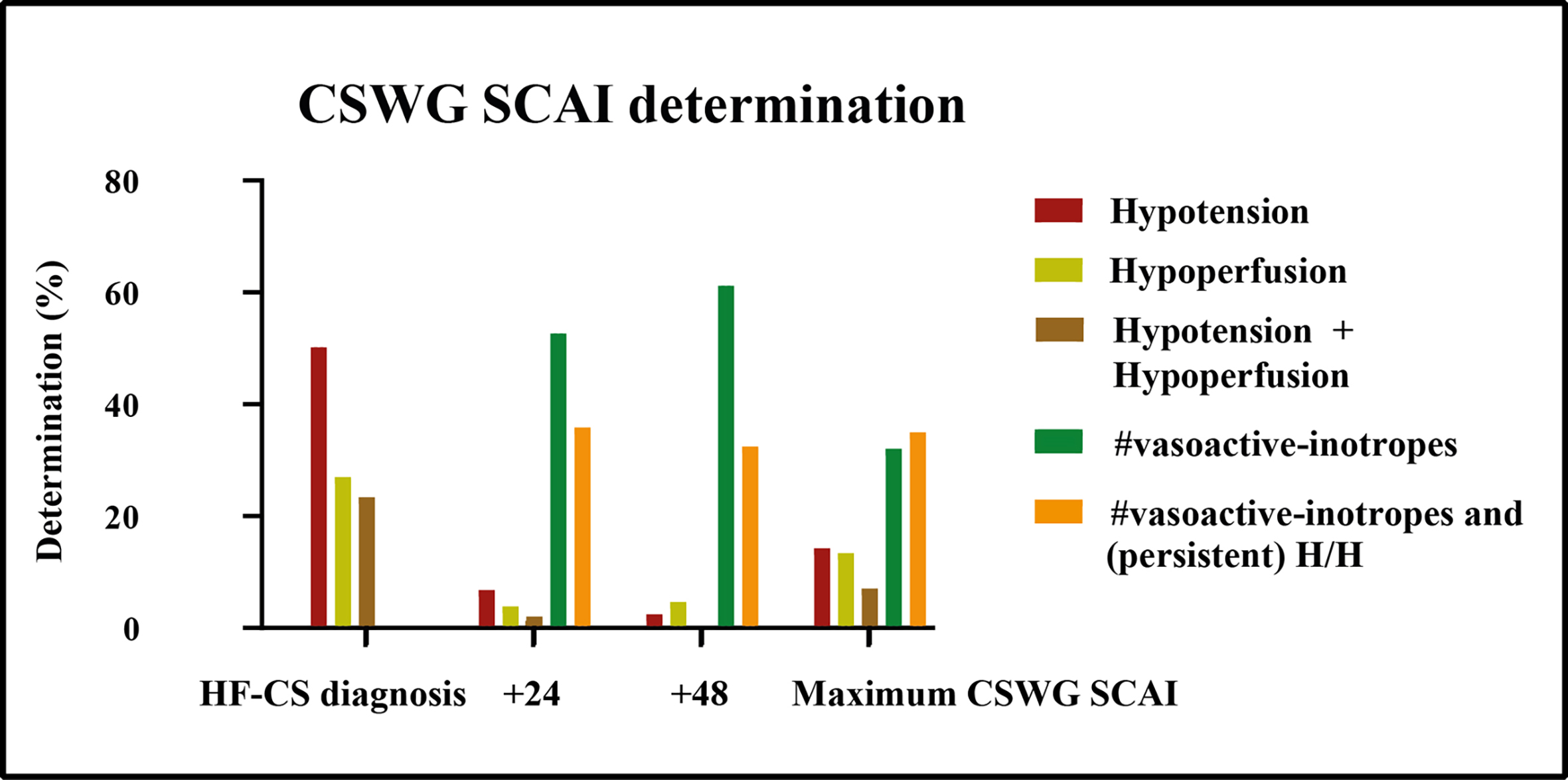

Supplement: Supplementary Fig. 1 — CSWG-SCAI determinants. Parameters used for CSWG-SCAI determination at each timepoint. Abbreviations: #: Number of, H/H: Hypotension/Hypoperfusion. [file mmc2.jpg]

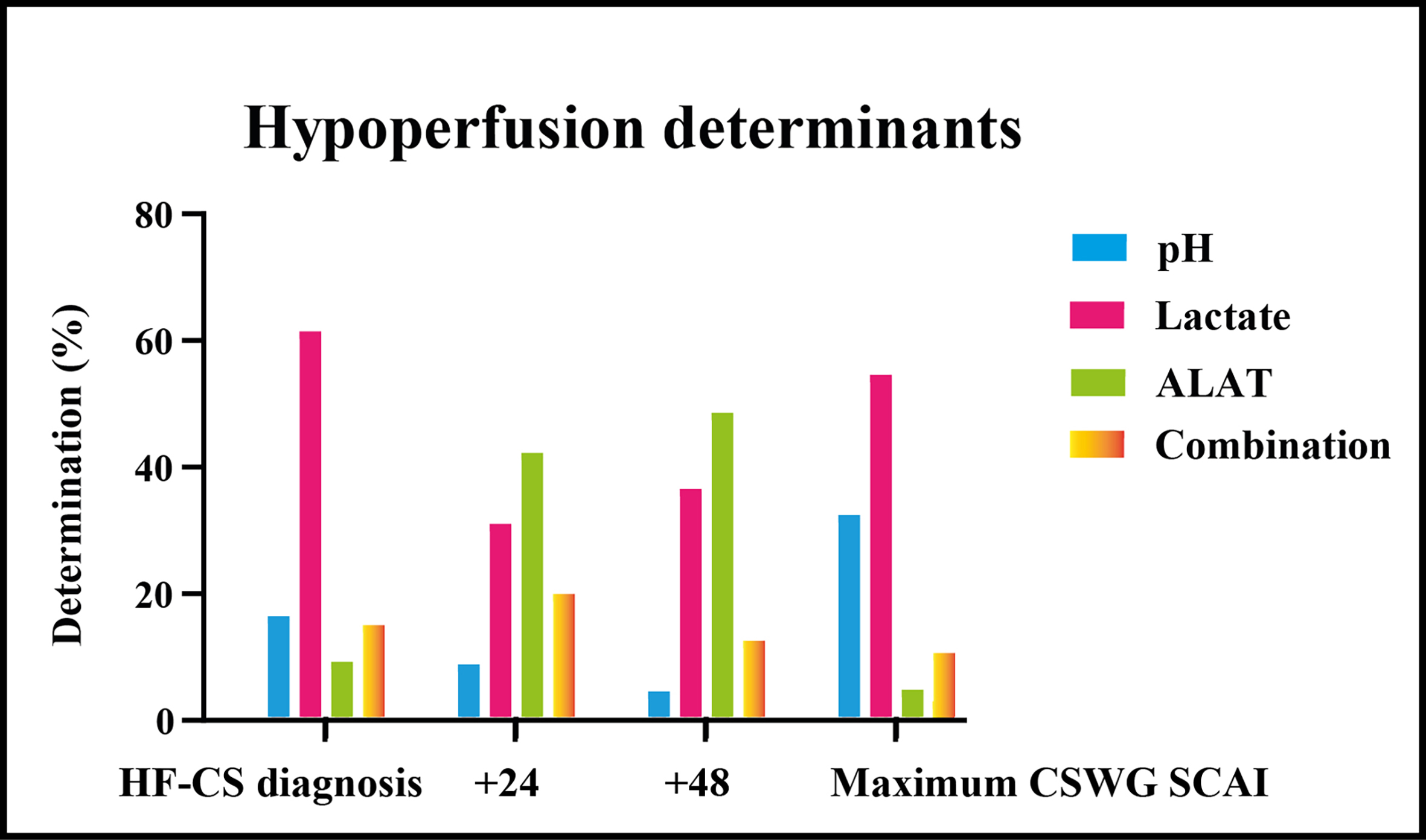

Supplement: Supplementary Fig. 2 — Hypoperfusion determinants for each timepoint. Abbreviations: CSWG-SCAI: Cardiogenic Shock Working Group modified Society for Cardiovascular Angiography & Interventions [file mmc3.jpg]

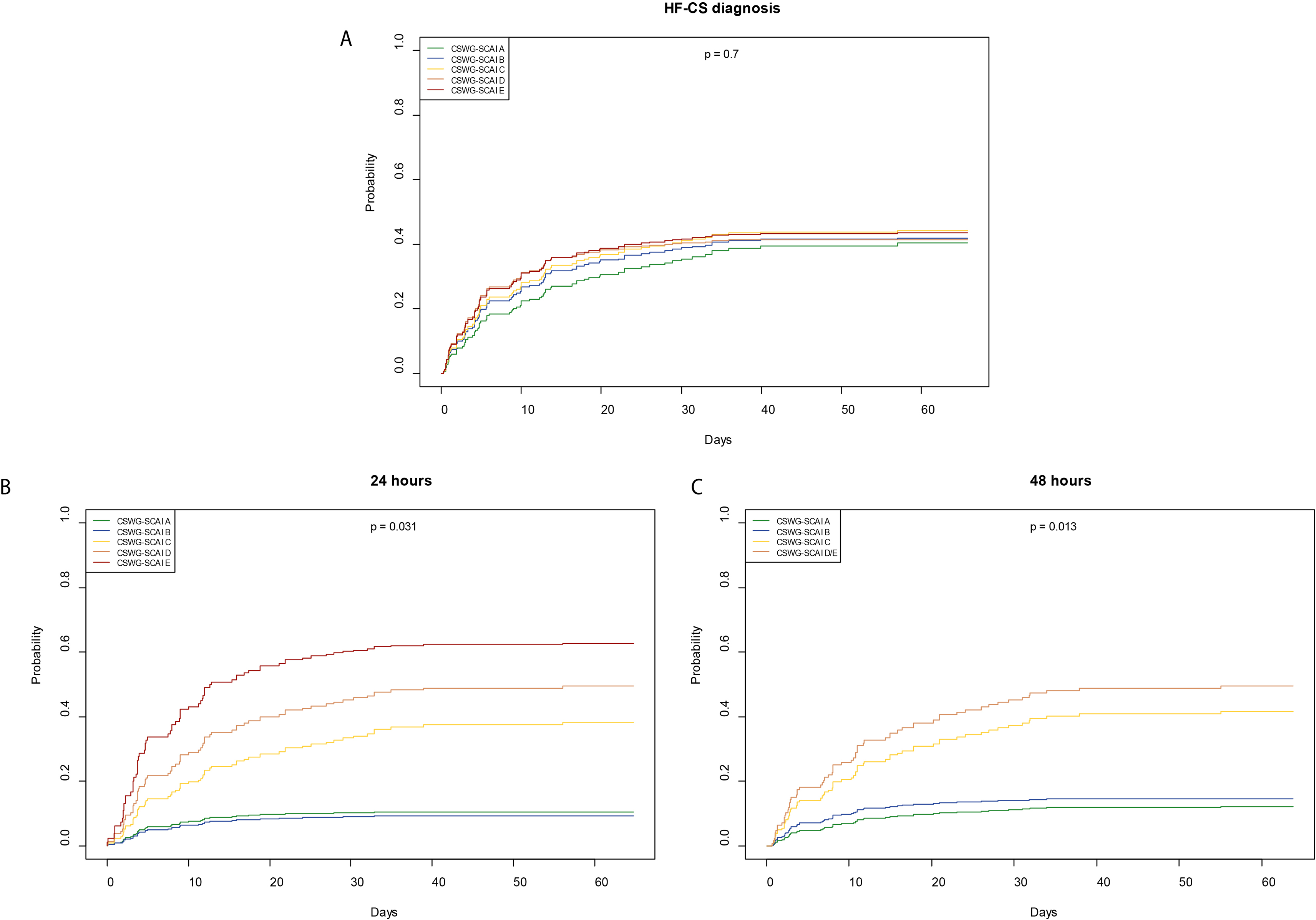

Supplement: Supplementary Fig. 3 — Time-to-event analysis CSWG-SCAI and in-hospital mortality (A) HF-CS diagnosis (B) at 24 hours (C) at 48 hours [file mmc4.jpg]
