# Supplementary material for: Predictive value of the cardiogenic shock working group-modified SCAI criteria in early-stage heart failure-related cardiogenic shock
Source: Int J Cardiol Heart Vasc. 2025 Aug 26;60:101776. doi: 10.1016/j.ijcha.2025.101776 (PMC12398876; doi:10.1016/j.ijcha.2025.101776)
Supplement: Supplementary Data 1 [file mmc1.docx]

# Supplemental tables

**Supplemental table 1.** Cutoff values of hypotension, hypoperfusion, and treatment intensity parameters used for CSWG-SCAI stage determination.

|  | **CSWG-SCAI A** | **CSWG-SCAI** **B** | **CSWG-SCAI C** | **CSWG-SCAI D** | **CSWG-SCAI E** |
| --- | --- | --- | --- | --- | --- |
| **Hypotension** |  |  |  |  |  |
| SBP, mmHg | > 90 | 60 – 90 | 60 – 90 | 60 – 90 | <60 |
| MAP, mmHg | > 65 | 55 – 65 | 55 – 65 | 55 – 65 | <50 |
|  |  | **OR** | **AND** | **AND** | **OR** |
| **Hypoperfusion** |  |  |  |  |  |
| pH | ≥ 7.2 | ≥ 7.2 | ≥ 7.2 | ≥ 7.2 | <7.2 |
| Lactate, mmol/L | <2 | 2 – 5 | 2 – 5 | 5 – 10 | >10 |
| ALAT, U/L | < 200 | 200 – 500 | 200 – 500 | >500 | Not defined |
| **Vasoactive-inotropic support and MCS** | No drugs  No devices | No drugs  No devices | 1 drug/device  without hypotension or hypoperfusion | Total of 2-5 drugs or devices  **OR**  1 drug/device with persistent hypotension or hypoperfusion | ≥3 drugs/devices |
| **Out-of-hospital cardiac arrest** | No | No | No | No | Yes |
| **SCAI-UO** |  |  |  |  |  |
|  | **AND** | **OR** | **AND** | **OR** | **OR** |
| Urine output (ml/kg/h) | ≥0.5 | ≤0.5 | ≤0.5 | ≤0.3  AND SBP ≥90  AND MAP ≥65 | ≤0.3  AND SBP ≤90  OR MAP ≤65 |

Abbreviations: CSWG-SCAI: Cardiogenic Shock Working Group modified Society for Cardiovascular Angiography & Interventions, SBP: Systolic blood pressure, MAP: Mean arterial pressure, ALAT: Alanine aminotransferase, MCS: Mechanical circulatory support, UO: Urine output.

**Supplemental table 2.** Univariate and multivariate predictors for in-hospital mortality at each timepoint.

| **Predictors** | **Univariate p-level** |  |
| --- | --- | --- |
| Age | **0.003** |  |
| Sex | 0.983 |  |
| BMI | 0.275 |  |
| Admission Hb | **0.004** |  |
| Hypertension history | 0.646 |  |
| Diabetes mellitus | 0.759 |  |
| ICD | 0.960 |  |
| Heart failure de novo | 0.536 |  |
| Chronic kidney disease | 0.387 |  |
| AKI at admission | 0.233 |  |
| eGFR at admission | **0.001** |  |
| Noradrenaline onset HF-CS | 0.862 |  |
| Enoximone onset HF-CS | 0.323 |  |
| Milrinone onset HF-CS | 0.706 |  |
| Dobutamine onset HF-CS | 0.553 |  |
| Dopamine onset HF-CS | 0.645 |  |
| **CSWG-SCAI** |  | **Multivariate p-level** |
| CSWG-SCAI HF-CS diagnosis | 0.994 | 0.995 |
| CSWG-SCAI +24 | **0.005** | **0.039** |
| CSWG-SCAI +48 | **0.005** | **0.024** |
| Maximum CSWG-SCAI | **<0.001** | **<0.001** |
| **Urine output** |  | **Adjusted for CSWG-SCAI** |
| UO HF-CS diagnosis | **<0.001** | **<0.001** |
| UO +24 | **<0.001** | **<0.001** |
| UO +48 | **0.002** | **0.013** |
| UO maximum SCAI-UO | **0.001** | **0.001** |

Multivariate adjustment was performed for linear effects of continuously measured age (in years) and Hb (in mmol/L).

Abbreviations: BMI: Body mass index, ICD: Implantable cardioverter-defibrillator, UO: Urine output

**Supplemental table 3.** Hospitalization duration stratified per CSWG-stage for each timepoint

|  | **HF-CS diagnosis** | **+24 hours** | **+48 hours** | **Maximum SCAI-UO** |
| --- | --- | --- | --- | --- |
| **CSWG-SCAI** | Hospitalization duration (days) | Hospitalization duration (days) | Hospitalization duration (days) | Hospitalization duration (days) |
| **A** | 17 (9-30) | 9 (4-23) | 14 (6-27) | NA |
| **B** | 14 (6-23) | 12 (5-16) | 12 (7-18) | NA |
| **C** | 14 (8-24) | 20 (13-28) | 20 (13-27) | 16 (8-27) |
| **D** | 13 (5-24) | 13 (6-23) | 14 (8-23) | 17 (9-24) |
| **E** | 7 (4-23) | 7 (5-22) | 21 (4-*) | 12 (5-23) |
| **P-value** | **0.034** | **<0.001** | **0.036** | **0.022** |

Values are presented as median (IQR). Comparison between the CSWG-SCAI classifications was performed using the Kruskal-Wallis test.
* n=2

**Supplemental table 4.** Predictive value for in-hospital mortality for each individual CSWG-SCAI component

|  | **HF-CS diagnosis** | **+24 hour** | **+48 hour** | **Maximum CSWG-SCAI** |
| --- | --- | --- | --- | --- |
|  | **OR** | **OR** | **OR** | **OR** |
| **Hypotension** |  |  |  |  |
| SBP ≤90 mmHg | 1.359 (p=0.279) | 2.429 (p=0.018) | 2.423 (p=0.054) | 1.549 (p=0.128) |
| SBP <60 mmHg | 2,133 (p=0.412) | * | ** | 3.592 (p=0.070) |
| MAP ≤65 mmHg | 1,756 (p=0.052) | 1.722 (p=0.141) | 1.228 (p=0.672) | 1.644 (p=0.087) |
| MAP <50 mmHg | 1,447 (p=0.433) | 1.648 (p=0.621) | ****** | 1.786 (p=0.135) |
| **Hypoperfusion** |  |  |  |  |
| pH <7.2 | 0.700 (p=0.590) | 0.700 (p=0.590) | * | 0.545 (p=0.203) |
| Lactate ≥2 mmol/L | 0.511 (p=0.139 | 1.583 (p=0.428) | 2.000 (p=0.350) | 0.621 (p=0.810) |
| Lactate ≥5 mmol/L | 0.677 (p=0.454) | * | 2.000 (p=0.589) | 1.029 (p=0.947) |
| Lactate ≥10 mmol/L | 0.000 (p=0.999) | * | * | 1.341 (p=0.690) |
| ALAT ≥200 U/L | 1.043 (p=0.937) | 1.050 (p=0.936) | 0.308 (p=0.167) | 1.400 (p=0.562) |
| ALAT ≥500 U/L | 0.566 (p=0.439) | 0.487 (p=0.272) | 0.182 (p=0.069) | 1.389 (p=0.615) |
| **Number of vasoactive-inotropes and MCS** | NA | p=0.006 | p <0.001 | P=0.012 |
| 1 | NA | 9.079 (p <0.001) | 5.233 (p <0.001) | 0.319 (p=0.020) |
| 2 | NA | 8.250 (p=0.002) | 5.880 (p <0.001) | 0.481 (p=0.161) |
| 3+ | NA | 11.000 (p=0.119) | NA | 2.462 (p=0.332) |
| **Persistent hypotension/hypoperfusion** | NA | 8.593 (p <0.001) | 3.834 (p=0.003) | 6.592 (p=0.001) |
| **Out-of-hospital cardiac arrest** | NA | NA | NA | NA |

***** All patients died (n=1)

****** No values below/above threshold available

**Supplemental table 5.** Comparison of patients with and without the availability of UO

|  | **HF-CS diagnosis** | | **+24 hours** | | **+48 hours** | | **Maximum SCAI-UO** | |
| --- | --- | --- | --- | --- | --- | --- | --- | --- |
| **UO available?** | **No** | **Yes** | **No** | **Yes** | **No** | **Yes** | **No** | **Yes** |
| **Characteristics** |  |  |  |  |  |  |  |  |
| Age | 71 ±14 | 69 ±12 | 70 ±15 | 70 ±12 | 71 ±14 | 69 ±12 | 70 ±14 | 70 ±12 |
| Female | 48 (34) | 14 (22) | **35 (42)** | **27 (22)*** | **39 (41)** | **23 (21)*** | **43 (36)** | **19 (21)*** |
| BMI | 26 ±5 | 28 ±5 | 27 ±6 | 27 ±5 | 26 ±6 | 27 ±5 | 26 ±5 | 27 ±5 |
| De novo HF | **27 (19)** | **3 (5) *** | **18 (21)** | **12 (10)*** | **20 (21)** | **10 (9)*** | 22 (19) | 8 (9) |
| Ethiology HF |  | ***** |  | ***** |  | ***** |  | ***** |
| Ischemic | **54(38)** | **39 (60)** | **28 (33)** | **65 (52)** | **33 (34)** | **60 (54)** | **43 (36)** | **50 (56)** |
| Non-Ischemic | **86 (60)** | **26 (40)** | **53 (63)** | **59 (48)** | **60 (63)** | **52 (46)** | **72 (61)** | **40 (44)** |
| In-hospital mortality | 58 (41) | 29 (45) | 39 (47) | 48 (39) | 46 (48) | 41 (37) | **41 (35)** | **46 (51)*** |
| **CSWG-SCAI** |  |  |  |  |  |  |  |  |
| SBP, mmHg | 96 ±26 | 97 ±23 | 104 ±19 | 105 ±19 | 109 ±19 | 110 ±19 | 95 ±28 | 99 ±26 |
| MAP, mmHg | 71 ±18 | 72 ±17 | 74 ±13 | 76 ±13 | 78 ±11 | 80 ±12 | 70 ±20 | 71 ±18 |
| Lactate, mmol/L | 3.3 ±2.2 | 3.6 ±2.1 | 2.4 ±2.0 | 2.0 ±0.9 | 2.7 ±2.6 | 2.1 ±1.4 | 4.2 ±4.0 | 4.0 ±3.3 |
| ALAT, U/L | 209 ±228 | 228 ±258 | 585 ± 542 | 542 ±828 | 72 ±725 | 621 ±745 | 248 ±458 | 442 ±950 |
| pH | 7.36 ± 0.13 | 7.40 ±0.15 | 7.41 ±0.08 | 7.42 ±0.11 | 7.42 ± 0.10 | 7.39 ±0.10 | 7.32 ±0.15 | 7.36 ±0.15 |
| Number of vasoactives  /inotropes/MCS |  |  |  |  |  |  |  |  |
| 0 | NA | NA | 17 (24) | 19 (16) | 24 (32) | 29 (26) | 10 (9) | 11 (12) |
| 1 | 107 (75) | 55 (85) | 37 (52) | 78 (63) | 32 (43) | 65 (58) | 70 (59) | 68 (58) |
| 2 | 36 (25) | 10 (15) | 17(24) | 25 (20) | 18 (24) | 18 (16) | 36 (31) | 21 (23) |
| 3+ | 0 (0) | 0 (0) | 0 (0) | 2 (2) | 0 (0) | 0 (0) | 2 (2) | 8 (9) |
| Persistent hypotension /hypoperfusion | NA | NA | 13 (18) | 12 (10) | 11 (15) | 14 (13) | 8 (7) | 12 (13) |

Values are presented as mean ± SD, or absolute numbers (%). Abbreviations: HF: Heart failure, SBP: Systolic blood pressure, DBP: Diastolic blood pressure
(*) p <0.05.
(**) p <0.001

# Supplemental figure legends

**Supplemental figure 1. CSWG-SCAI determinants.** Parameters used for CSWG-SCAI determination at each timepoint. Abbreviations: #: Number of, H/H: Hypotension/Hypoperfusion.

**Supplemental figure 2. Hypoperfusion determinants for each timepoint.**Abbreviations: CSWG-SCAI: Cardiogenic Shock Working Group modified Society for Cardiovascular Angiography & Interventions

**Supplemental figure 3. Time-to-event analysis CSWG-SCAI and in-hospital mortality**

1. HF-CS diagnosis **(B)** at 24 hours **(C)** at 48 hours
